# Meteorological Table

**Published:** 1813-07

**Authors:** 


### METEOROLOGICAL TABLE.

### *From May 26, to June 25,* 1813.

| | D. | Therm. | | | Barom. | | Hygrom. Dry.——Damp. | | | Winds. | Atmos. Variation. |
|---|---|---|---|---|---|---|---|---|---|---|---|
| | 26 | 53 | 56 | 53 | 30 | — | 2 | 3 | 2 | NW | Hail...with Thun |
| | 27 | 54 | 57 | 53 | $30^1$ | — | 2 | 3 | 4 | W | C. F... |
| | 28 | 59 | 71 | 56 | 30 | — | 11 | 23 | 14 | SE | F.... |
| ● | 29 | 54 | 73 | 65 | | | 9 | 30 | 15 | W | F.... |
| | 30 | 64 | 68 | 63 | | | 15 | 12 | 8 | NW | C. R. C.. |
| | 31 | 66 | 76 | 63 | $30^1$ | — | 14 | 20 | 15 | W | F.... |
| | 1 | 69 | 76 | 66 | | | 15 | 31 | 16 | W | F.... |
| | 2 | 66 | 77 | 68 | 30 | — | 17 | 35 | 19 | SW | F.... |
| | 3 | 67 | 68 | 59 | $30^1$ | 2 | 16 | 18 | 15 | W | F.. C.. |
| | 4 | 61 | 70 | 60 | $30^1$ | — | 11 | 20 | 13 | W | C. F.. C.. |
| ◐ | 5 | 61 | 59 | 54 | 30 | $29^9$ | 16 | 10 | 12 | NE | F.. C.. |
| | 6 | 56 | 58 | 53 | $29^8$ | | — | 10 | 12 | NE | C... |
| | 7 | 53 | 71 | 57 | | | 11 | 26 | 18 | NE | C.. F... |
| | 8 | 60 | 74 | 58 | | | 15 | 37 | 20 | W | F.... |
| | 9 | 58 | 66 | 61 | $29^6$ | 5 | 17 | 12 | 6 | NW | F... |
| | 10 | 59 | 65 | 59 | $29^7$ | 8 | 15 | 36 | 24 | W | F... |
| | 11 | 58 | 68 | 61 | $29^9$ | — | 19 | 37 | 31 | SW | F... |
| | 12 | 59 | 74 | 56 | 30 | — | 10 | 20 | 19 | W | F... |
| | 13 | 60 | 66 | 61 | $30^1$ | 30 | 20 | 25 | 15 | W | F.. C.. |
| ○ | 14 | 59 | 64 | 56 | 30 | $29^9$ | 10 | 12 | 11 | W | F... R. C.. |
| | 15 | 57 | 71 | 56 | $29^9$ | — | 10 | — | 9 | W | R.. F. R... F. |
| | 16 | 55 | 69 | 54 | 30 | — | 12 | 30 | 25 | NW | F.. |
| | 17 | 55 | 68 | 54 | | | 20 | 24 | 15 | S | F.. |
| | 18 | 55 | 61 | 52 | $30^1$ | — | 20 | — | 19 | E | C. F..C.. |
| | 19 | 54 | 57 | 53 | | | 20 | 19 | 15 | E | C.. |
| | 20 | 58 | 60 | 56 | $30^2$ | — | 15 | — | 16 | NE | F.. C.. |
| ◑ | 21 | 54 | 65 | 53 | | | 20 | 25 | 22 | NW | F.. |
| | 22 | 57 | 68 | 51 | $30^3$ | — | 15 | 26 | 19 | NE | F.. |
| | 23 | 60 | 72 | 63 | $30^3$ | $30^2$ | 26 | 37 | 30 | NE | F.... |
| | 24 | 58 | 70 | 57 | $0^3$ | — | 23 | 25 | 15 | NE | F... |
| | 25 | 57 | 71 | 59 | | | 20 | 34 | 16 | NE | F.. |

Quantity of rain from the 26th of May to the 25th of June, $\frac{44}{100}$ of an inch.

This interval has been remarkably dry; the mercury has been 23 days steadily at 30 or above; the prevailing winds have blown from the N. and NE.; and the temperature has been low. Some catarrhal affections, instances of inflammatory angina, and pneumonic inflammation, have occurred in private practice; but the records of public dispensaries indicate that this interval has been unusually free from disease.

*Prince's Street, Cavendish Square.*

MONTHLY

